# Passage through the Ocular Barriers and Beneficial Effects in Retinal Ischemia of Topical Application of PACAP1-38 in Rodents

**DOI:** 10.3390/ijms18030675

**Published:** 2017-03-21

**Authors:** Dora Werling, William A. Banks, Therese S. Salameh, Timea Kvarik, Laszlo Akos Kovacs, Alexandra Vaczy, Edina Szabo, Flora Mayer, Rita Varga, Andrea Tamas, Gabor Toth, Zsolt Biro, Tamas Atlasz, Dora Reglodi

**Affiliations:** 1 Department of Anatomy, University of Pecs, Medical School, Pecs 7624, Hungary; doraw86@gmail.com (D.W.); kvarik.timi@gmail.com (T.K.); laszlo.akos.kovacs@aok.pte.hu (L.A.K.); vaczyalexandra@gmail.com (A.V.); szaboedina90@gmail.com (E.S.) ; mayerflo7@gmail.com (F.M.); ritusvarga@citromail.hu (R.V.); andreatamassz@gmail.com (A.T.); attam33@gmail.com (T.A.); 2 Department of Ophthalmology, University of Pecs, Pecs 7624, Hungary; biro.zsolt@pte.hu; 3 Geriatric Research, Education, and Clinical Center, Veterans Affairs Puget Sound Health Care System, Seattle, WA 98108, USA; wabanks1@uw.edu (W.A.B.); tsalameh@uw.edu (T.S.S.); 4 Division of Gerontology and Geriatric Medicine, Department of Medicine, University of Washington School of Medicine, Seattle, WA 98122, USA; 5 Department of Medical Chemistry, University of Szeged, Szeged 6720, Hungary; toth.gabor@med.u-szeged.hu; 6 Department of Sportbiology, University of Pecs, Pecs 7624, Hungary; 7 Janos Szentagothai Research Center, University of Pecs, Pecs 7624, Hungary

**Keywords:** eye drops, PACAP, ischemic retinopathy, carotid artery occlusion, neuroprotection

## Abstract

The neuropeptide pituitary adenylate cyclase activating polypeptide (PACAP) has two active forms, PACAP1-27 and PACAP1-38. Among the well-established actions are PACAP’s neurotrophic and neuroprotective effects, which have also been proven in models of different retinopathies. The route of delivery is usually intravitreal in studies proving PACAP’s retinoprotective effects. Recently, we have shown that PACAP1-27 delivered as eye drops in benzalkonium-chloride was able to cross the ocular barriers and exert retinoprotection in ischemia. Since PACAP1-38 is the dominant form of the naturally occurring PACAP, our aim was to investigate whether the longer form is also able to cross the barriers and exert protective effects in permanent bilateral common carotid artery occlusion (BCCAO), a model of retinal hypoperfusion. Our results show that radioactive PACAP1-38 eye drops could effectively pass through the ocular barriers to reach the retina. Routine histological analysis and immunohistochemical evaluation of the Müller glial cells revealed that PACAP1-38 exerted retinoprotective effects. PACAP1-38 attenuated the damage caused by hypoperfusion, apparent in almost all retinal layers, and it decreased the glial cell overactivation. Overall, our results confirm that PACAP1-38 given in the form of eye drops is a novel protective therapeutic approach to treat retinal diseases.

## 1. Introduction

Pituitary adenylate cyclase activating polypeptide (PACAP) is a well-known neuropeptide that acts on G protein-coupled receptors, namely PAC1, and VPAC1 as well as VPAC2 receptors. PACAP exists in two biologically active forms, PACAP1-27 and PACAP1-38. PACAP has a wide variety of diverse biological effects in different cells and tissues, from thermoregulatory effects [1], actions on cardiac excitability [2], thyroid gland functions [3], chondrogenesis [4] and muscle contraction [5]. Among the well-established and most intensively studied actions are PACAP’s neurotrophic and neuroprotective effects. PACAP has been shown to protect neurons against various noxious stimuli in vitro and in vivo [6,7,8] For example, PACAP is protective in traumatic brain [9] and spinal cord injury [10], in global and focal cerebral ischemia [6], in models of Huntington’s disease [11], and in Parkinson’s disease [12,13,14].

PACAP’s trophic and protective effects in the retina are also well-established and confirmed by several research groups. PACAP influences the development of the retina [15], and protects against several in vitro and in vivo retinal injuries [16,17,18,19]. In vitro, the peptide has been shown to protect whole retinal tissue cultures [20] and cell cultures from various cell types, including pigment epithelial cells [21,22] and retinal ganglion cells [23]. In vivo, PACAP is protective against NMDA-, glutamate- and kainate-induced retinal excitotoxicity [24,25,26], optic nerve transection [27], UV-induced retinal damage [28], diabetic retinopathy [29,30], retinopathy of prematurity [31] and ischemia-induced injury [32,33,34].

In vivo studies apply PACAP through intravitreal injection, whereby the peptide is injected into the vitreous body and reaches the retina by diffusion through the vitreous and layers of the retina itself. This route of administration of therapeutic agents is a common practice in the treatment of several retinopathies, including vascular endothelial growth factor (VEGF) antagonists in age-related macular degeneration and diabetic retinopathy. However, delivery of therapeutic substances in eye drops is more convenient and causes fewer side effects. The main problem with eye drops is that most substances do not cross the ocular barriers to reach a suitable therapeutic concentration in the retina. We have recently provided evidence that PACAP1-27, the shorter form of PACAP, crosses the ocular barriers and reaches the retina in an amount sufficient to exert retinal protection in a model of retinal ischemia [35]. In this study, we investigated several vehicles to deliver PACAP1-27 as eye drops, and found that benzalkonium-chloride (benzalkonium solution for ophthalmic use -SOCB) was the most effective vehicle. Using PACAP1-27 in benzalkonium solution eye drops in ischemic retinal injury, we demonstrated less pronounced thinning of the retinal layers, decreased neuronal loss, decreased ischemia-induced glial activation, reduced cytokine levels and an activation of protective anti-apoptotic molecules [35].

In our first study using PACAP in eye drops, we chose PACAP1-27 for its better lipid solubility, stability and under the assumption that it crossed ocular barriers more easily since it is 11 amino acids shorter than PACAP1-38 [36]. From the two biologically active forms of the peptide, PACAP1-38 is the more common form, comprising about 90% of PACAP in the mammalian body. They bind to the same receptors and have the same or similar actions in most models [37]. In models and experimental setups, where the two main forms of the peptide exert similar actions, PACAP1-38 is usually more potent than PACAP1-27, mainly explained with the stronger receptor-binding capacity of PACAP1-38 [38,39]. However, several differences also exist between the two peptides in some other models, including opposite effects on luteinizing hormone (LH) secretion [40]. Therefore, the aim of the present study was to investigate whether PACAP1-38, the longer form of the peptide, is also able to cross the ocular barriers and exert retinoprotective effects in permanent bilateral common carotid artery occlusion (BCCAO), a model of retinal hypoperfusion.

## 2. Results

### 2.1. Retina Morphology and Morphometry

**Figure 1:**
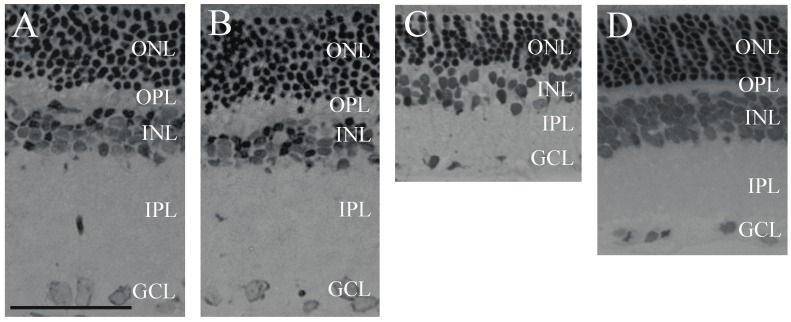
Light microphotographs of retinal sections. Retinal tissue from bilateral common carotid artery occlusion (BCCAO) (*n* = 9) (**C**) showed severe degeneration compared to sham (*n* = 3) (**A**) and sham + PACAP1-38 eye drops retinas (*n* = 3) (**B**); The retinal layers of BCCAO rats following treatment with eye drops containing PACAP1-38 eye drops (*n* = 9) (**D**) showed only mild degeneration. (Scale bar: 50 µm). Abbreviations: ONL: outer nuclear layer, OPL: outer plexiform layer, INL: inner nuclear layer, IPL: inner plexiform layer, GCL: ganglion cell layer.

**Figure 2:**
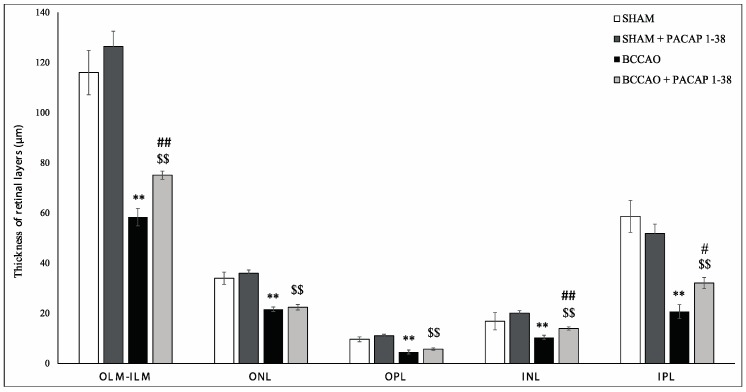
Quantification of retinal layers in sham (*n* = 3 sham, *n* = 3 sham + PACAP1-38 eye drops) and BCCAO (*n* = 9 BCCAO, *n* = 9 BCCAO + PACAP1-38 eye drops) animals: the right eye was treated with PACAP1-38 eye drops dissolved benzalkonium solution, the left eye served as a control receiving only benzalkonium solution. Comparison of retinal layers in sham animals with control, BCCAO rats and those receiving PACAP1-38 eye drops after carotid occlusion. Abbreviations: OLM, outer limiting membrane; ILM, inner limiting membrane; ONL, outer nuclear layer; OPL, outer plexiform layer; INL, inner nuclear layer; IPL, inner plexiform layer. Morphometric analysis showed that treatment with eye drops improves the structure of all the retinal layers (except ONL and OPL). Data are expressed as mean ± SEM. Statistical significance (** *p* < 0.001 vs. sham retinas, ^$$^ *p* < 0.001 vs. sham + PACAP1-38 retinas, ^#^ *p* < 0.05; ^##^ *p* < 0.001 vs. BCCAO retinas) was calculated by two-way ANOVA followed by Fischer’s post hoc test.

**Figure 3:**
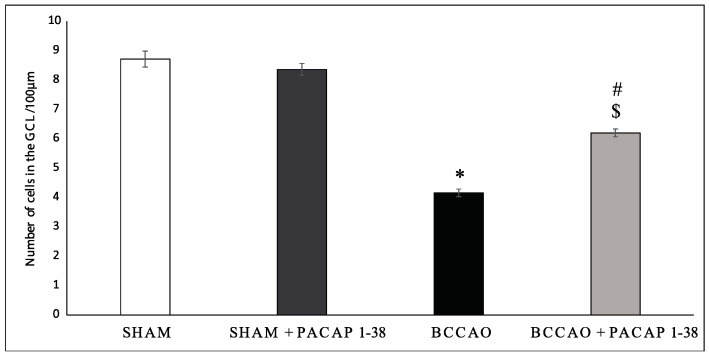
Number of cells in the ganglion cell layer (GCL)/100 µm length. Data are expressed as mean number of cells/100 µm GCL length ± SEM. Statistical significance (* *p* < 0.05 vs. sham retinas, ^$^ *p* < 0.05 vs. sham + PACAP1-38 retinas, ^#^ *p* < 0.05 vs. BCCAO retinas) was calculated by two-way ANOVA followed by Fischer’s *post hoc* test.

BCCAO resulted in severely reduced thickness of retinal layers two weeks after ligation, compared to sham retinas (Figure 1A,C). PACAP1-38 alone in sham animals did not result in changes in any of the retinal layers (Figure 1A,B). PACAP1-38 dissolved in solutio ophthalmica cum benzalkonio (briefly, benzalkonium) led to significant protection in the retina in BCCAO-lesioned retinas (Figure 1D); retinas treated with PACAP1-38 eye drops had preserved structure compared to control retinas (Figure 1D). OLM–ILM (outer limiting membrane–inner limiting membrane) distance was reduced by 49.7% (*p* < 0.001) in BCCAO retinas compared to sham controls, but it was only 40.6% (*p* < 0.001) in the eyes treated with PACAP1-38 eye drops. A protection to a similar degree was found in the inner nuclear layer (INL) (BCCAO: 38.5%, PACAP1-38: 30.5%; *p* < 0.001), and inner plexiform layer (IPL) (BCCAO: 64.8%, PACAP1-38: 38.2%; *p* < 0.05), while no statistically significant attenuation of the damage was observed in the outer nuclear layer (ONL) (BCCAO: 36.5%, PACAP1-38: 37.7%) or outer plexiform layer (OPL) (BCCAO: 53.0%, PACAP1-38: 48.2%) (Figure 2). The number of cells in the ganglion cell layer (GCL) was significantly decreased after BCCAO by 52.4% (*p* < 0.05) and was significantly ameliorated by PACAP1-38 eye drops (decreased by 25.9%; *p* < 0.05) (Figure 3).

### 2.2. PACAP1-38 Uptake after Ocular Administration

**Figure 4:**
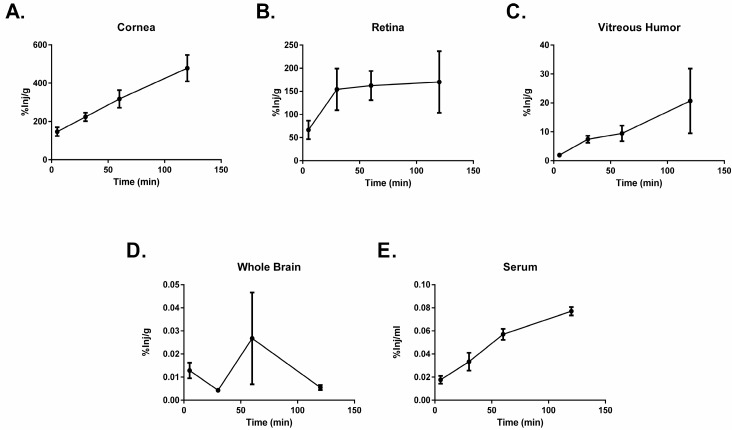
Distribution of ^125^I-PACAP1-38 after ocular administration. An amount of 1 × 10^6^ counts per minute (cpm) of radioactively labeled PACAP1-38 was administered ocularly to male CD-1 mice. The appearance of 125I-labeled PACAP1-38 in the cornea (**A**); retina (**B**); vitreous humor (**C**); whole brain (**D**); and serum (**E**) was measured at 5, 30, 60, and 120 min post application. Values are expressed as mean ± SEM for an *n* = 3 mice/time point.

An amount of 1 × 10^6^ counts per minute (cpm) of radioactively labeled PACAP1-38 was administered ocularly to male CD-1 mice. Mice were sacrificed at time points between 5 and 120 min post administration. The eye was dissected into the cornea, retina, and vitreous humor, and the brain and blood were collected. Levels of radioactively labeled PACAP1-38 were measured in each tissue for this analysis. Transport of PACAP1-38 occurred rapidly as detection of radioactivity was present in the eye, the brain, and the serum five minutes after application. PACAP1-38 displayed increasing transport across the cornea over the time course of the study (Figure 4). The vitreous body showed a similar profile to the cornea (Figure 4C). In the retina (Figure 4B), PACAP1-38 uptake increased to 30 min and plateaued. PACAP1-38 was detected after 5 min in the whole brain (Figure 4D) and levels remained stable throughout the observation time. PACAP1-38 showed increased clearance into the blood stream after 120 min (Figure 4E).

### 2.3. PACAP1-38 Stability after Ocular Administration

To determine the extent to which the radioactivity observed in the regions of the eye, brain, and serum after ocular administration of ^125^I-PACAP1-38 accurately represents the presence of the administered protein, we performed an acid precipitation on the tissue. This was completed at 5 and 60 min post application. The radioactivity of each sample after precipitation is reported as a percentage of a matched processing control (Table 1). No statistical differences were observed between the two time points (*p* > 0.05 by two-way analysis of variance) in any of the samples. In the eye, PACAP1-38 is about 50% intact, while in the brain the levels are higher (10 min: 104.71 ± 17.72; 60 min: 75.09 ± 41.13). This indicates that ocularly administered ^125^I-PACAP1-38 reaches all tissues intact and then degradation occurs. We can postulate that the brain has fewer degrading enzymes than the eye or the blood. In the serum, ocularly administered ^125^I-PACAP1-38 is almost completely degraded by 5 min. This is not surprising because the half-life of PACAP injected into mice and humans is between 2 and 10 min due to enzymatic degradation. This indicates that we will not obtain off-target effects from PACAP1-38 as it degrades rapidly in the blood stream.

### 2.4. Immunohistochemical Analysis of Retinal Glial Cell Activation

**Figure 5:**
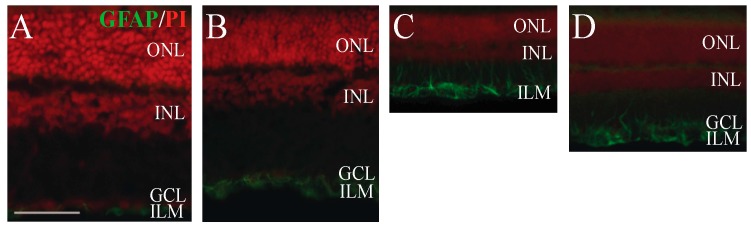
Representative retinal sections stained by GFAP antibody showing the effect of BCCAO (*n* = 4 BCCAO, *n* = 4 BCCAO + PACAP1-38) compared with sham (*n* = 4 sham, *n* = 4 sham + PACAP1-38 eye drops) retinas. PI was used to detect the nuclear components. In the sham (**A**) and sham + PACAP1-38 (**B**) retina preparations GFAP-immunoreactivity was restricted to the inner part and internal endfeet of Müller cells. Retinal degeneration caused by BCCAO (**C**) showed significant upregulation of immunoreactivity (*p* < 0.001). After PACAP1-38 eye drops (**D**), the immunopositivity was significantly reduced (*p* < 0.05). Abbreviations: ONL, outer nuclear layer; INL, inner nuclear layer; GCL, ganglion cell layer; ILM, inner limiting membrane; PI, propidium iodide; GFAP, glial fibrillary acidic protein. (Scale bar: 50 µm).

Müller glial cells are specific glial cells of the retina. Their cell bodies are found in the INL, and their endfeet extend between OLM and ILM. Glial fibrillary acidic protein (GFAP) filaments are intermediate filaments expressed mainly by astrocytes and ependymal cells in the central nervous system, and are also present in the inner part of the Müller glial cells (Figure 5A,B) [41]. GFAP signal shows upregulation after various injuries, showing over-activation of the Müller glial cells. Similar to earlier observations, we could detect significant upregulation of the GFAP immunostaining (*p* < 0.001) in the retina sections after BCCAO (Figure 5C). Similar to our recent study, an immunopositive signal was observed not only in the inner endfeet, but also in the entire cell extending from the OLM to ILM (Figure 5C). PACAP1-38 eye drops significantly (*p* < 0.05) attenuated the GFAP upregulation (Figure 5D).

## 3. Discussion

In the present study, we demonstrated that PACAP1-38, delivered as an eye drop, effectively counteracted the deleterious effects of rat retinal ischemia induced by permanent ligation of both carotid arteries. The present results are in accordance with our recent observations with PACAP1-27 eye drops [35]. In this recent study, we confirmed that PACAP1-27, dissolved in benzalkonium-chloride, passes through the mouse ocular barriers from the corneal surface to the retina, and ameliorates the retinal lesion. Our present results show similar actions of PACAP1-38: the thinning of the retinal layers as well as the reduction of the cells in the ganglion cell layer was not as expressed after PACAP1-38 eye drops treatment as in the control animals. Furthermore, the over-activation of the Müller glial cells was attenuated. Studying the passage through the ocular barriers confirmed that PACAP could be detected in the cornea, vitreous body and then in the retina in a concentration high enough to reach retinal protection. PACAP could also be detected in the serum and brain, but levels were negligible in comparison to ocular levels.

Our present study, together with the previous study on PACAP1-27, highlights the potential therapeutic use of PACAP in the form of eye drops to treat retinal diseases. The novelty of our present results is that PACAP1-38, the most commonly occurring form of the peptide, exerts a retinoprotective effect similarly to PACAP1-27. Although most actions of PACAP1-27 and PACAP1-38 are similar or the same, several differences are also known. In most studies, PACAP1-27 binds to the receptors with less affinity, leading to less well-pronounced effects at the same concentration as PACAP1-38 [6,36]. The metabolic stability is also different: while PACAP1-38 is rapidly degraded in the serum by dipeptidyl-peptidase, accounting for the short half-life of the peptide, PACAP1-27 is more stable due to the resistance against dipeptidyl-peptidase reaction [36]. Penetration through membranes also differs: it has been demonstrated in a recent study that cell-penetrating PACAP1-38, in high concentrations, reduced viability of retinoblastoma cells, but PACAP1-27 had negligible effects [42]. Even opposite actions have been described: intracerebroventricular administration of PACAP1-38 led to an inhibition of the LH surge, while opposite reaction was observed after PACAP1-27 injection [40]. In the present study, we found that the retinoprotective efficacy of PACAP1-38 and PACAP1-27 were the same, and they both were able to cross the ocular barriers given in the form of eye drops.

In the retina, we demonstrated that intravitreal injection of PACAP1-27 and 1-38 led to the same degree of retinoprotection in monosodium glutamate-induced excitotoxic lesions [43]. In contrast, the receptor antagonists PACAP6-38 and PACAP6-27 further increased the excitotoxic damage, indicating the protective effect of the endogenous peptide [43], later confirmed in PACAP-deficient mice [44]. Peptides in the same peptide family as PACAP (VIP/secretin/glucagon peptide family) do not have such marked protective effects in the retina. VIP, the peptide with the closest structural similarity to PACAP, led to a neuroprotective effect in ischemic retinopathy at concentrations ten times higher than required for PACAP to exert the same degree of protection [45]. In a recent study, we compared the retinoprotective efficacy of glucagon and secretin, two other peptides from the same peptide family: they do not exert protective effects in ischemic retinal lesion, similarly to several other shorter fragments of PACAP tested [34].

PACAP eye drops have been used in previous experiments to treat corneal diseases. Nakamachi and coworkers [46] have recently described that PACAP eye drops stimulated tear secretion, increased cAMP release and aquaporin expression in the infraorbital lacrimal gland in mice and suppressed corneal keratinization in PACAP null animals. PACAP eye drops have also been shown to enhance corneal wound healing [47] and accelerate regeneration of corneal nerve endings [48]. However, our studies are the first to show that PACAP administered as eye drops can reach the retina and exert protective effects.

The protective mechanism is supposedly similar to what we have previously found with PACAP1-27 eye drops and with intravitreal injections of PACAP in different retinal injury models [17]. We have described that PACAP1-27 eye drops decreased retinal inflammatory cytokine expression and increased the protective Akt and ERK1/2 in hypoperfused retinas [35]. In other studies, we have provided evidence for the anti-apoptotic effects of the peptide in retinal lesions, including attenuation of caspases, JNK, p38, apoptosis-inducing factor, cytochrome c release, while increasing 14-3-3 protein expression and ERK, Bcl-XL and bad phosphorylation [49,50,51]. In summary, our present study confirms the therapeutic potential of PACAP eye drops in ischemic retinal lesion and provides further evidence for the protective efficacy of PACAP in neuronal ischemia [52,53,54].

## 4. Materials and Methods

### 4.1. Surgery and PACAP1-38 in Benzalkonium Solution Treatment

Wistar rats (*n* = 20:*n* = 12 for histological analysis, *n* = 8 for immunohistochemical analysis) weighing 250–300 g were subjected to permanent ligation of both common carotid arteries. The animals were bred and kept in the Animal Facility of the University of Pecs, Medical School. They were fed and watered ad libitum, under light/dark cycles of 12/12 h. All procedures were in accordance with institutional guidelines (ethical permission No: BA02/2000-31/2011, University of Pecs). Under isoflurane anesthesia, both common carotid arteries were exposed through a midline cervical incision and ligated with a 3-0 filament. Directly after the operation within 1 min, the right eye was treated with PACAP1-38 eye drops (1 µg/drop) (PACAP was synthesized at the Department of Medical Chemistry, University of Szeged, Szeged, Hungary). The vehicle used was benzalkonium-chloride in a concentration of 0.005%, as it was the most effective vehicle to achieve neuroprotection with PACAP1-27 eye drops, proven in our previous study [35]. The left eye served as a control, treated only with the vehicle. A group of animals served as the sham-operated group that underwent anesthesia and all steps of the surgical procedure except ligation of the carotid arteries. Rats were treated twice a day with one drop, for 5 consecutive days [35].

### 4.2. Histological Analysis of PACAP1-38 Eye Drops

Rats (*n* = 3 sham, *n* = 3 sham + PACAP1-38, *n* = 9 BCCAO, *n* = 9 BCCAO + PACAP1-38 retinas) were sacrificed with an overdose of anesthetic 2 weeks after BCCAO and eyes were processed for histological analysis. Histological analysis was performed as described previously [35]. Briefly, removed retinas were dissected in phosphate buffered saline (PBS), fixed in 4% paraformaldehyde dissolved in 0.1 M phosphate buffer (PB), embedded in Durcupan ACM resin, and two-µm-thick sections were stained with toluidine blue (Sigma, Budapest, Hungary). Four tissue blocks obtained from at least three rats were prepared and central retinal areas within 1 mm from the optic nerve were used (*n* = 5 measurements from one tissue block). The following parameters were measured on digital photographs taken with a Nikon Eclipse camera using the Spot program: cross-section from the outer limiting membrane (OLM) to the inner limiting membrane (ILM), and width of all retinal layers (outer nuclear layer-ONL, outer plexiform layer-OPL, inner nuclear layer-INL, inner plexiform layer-IPL). We also counted the number of cells in the ganglion cell layer (GCL). Results are presented as mean ± SEM. Statistical comparisons were made using the two-way ANOVA followed by Fischer’s post hoc analysis.

### 4.3. Analysis of the Passage of PACAP1-38 Eye Drops through the Ocular Barriers

PACAP1-38 was labeled with 125I using the lactoperoxidase method, as previously described with PACAP1-27 [35]. Briefly, 10 µg of PACAP1-38 (dissolved in 0.25 M chloride free phosphate buffer) was mixed with 30 µL of 0.4 M Na acetate (pH 5.6), 10 µL of lactoperoxidase (10 µg/mL dissolved in 0.1 M Na acetate pH 5.6), and 2 mCi of 125I. The reaction was started by adding 10 µL 30% H_2_O_2_ solution (prepared by adding 2 µL H_2_O_2_ in 30 ml deionized (dI) H_2_O). Ten minutes later, an additional 10 µL of the 30% H_2_O_2_ solution was added and the reaction was allowed to progress 10 min more. At the end of this second 10 min incubation, the reaction solution was purified with high performance liquid chromatography (HPLC, Shimadzu USA Manufacturing Inc., Columbia, MD, USA) using a C18 column (P.J. Cobert Associates, Inc., St. Louis, MO, USA).

The PACAP1-38 fraction collected from the HPLC was evaporated overnight in a fume hood using constant airflow to evaporate off the 0.1% trifluoroacetic acid in methanol solution that the fraction was collected in. The dried fraction was resuspended in 1% BSA Lactated Ringers’ solution and an acid precipitation was completed to assess the purity of the PACAP1-38 fraction. The acid precipitation was completed with 15% trichloroacetic acid. The percent of radioactivity precipitated was calculated using the following formula: 100 × [(CPMpellet)/(CPMpellet + CPMsupernatant)]. Only fractions that showed >90% activity in the precipitate were used in the experiment. A 1 × 10^6^ cpm solution was prepared in a 10 µL volume of benzalkonium-chloride. A 10 µL drop was placed on each eye delivering 1 × 10^6^ cpm per eye. The two eyes were pooled together for analysis.

An amount of 1 × 10^6^ cpm/eye in benzalkonium-chloride of radioactively labeled PACAP1-38 was administered once ocularly to male CD-1 mice. Mice were sacrificed at time points between 5 and 120 min post administration (5, 30, 60, and 120 min). The eye was dissected into the cornea, retina, and vitreous humor, and the brain and blood were collected from the carotid artery, and the eyes and whole brain removed. Levels of radioactively labeled PACAP1-38 were measured in each tissue for this analysis. Mice (*n* = 3) were used at each time point. The collected whole blood was allowed to clot at room temperature, centrifuged at 5400× *g* for 10 min at 4 °C, and 50 µL of the resulting serum was removed for use. The whole brain was collected and the eye dissected into the cornea, retina, and vitreous humor. The level of radioactivity in each of the regions and the serum was measured in a Wizard2 Automatic Gamma Counter (PerkinElmer, Waltham, MA, USA). The percentage of injected dose present in a milliliter of serum (%Inj/mL) and the percentage of the injected dose taken up per gram of tissue (%Inj/g) was calculated as previously described [55,56].

### 4.4. In Vivo Stability of ^125^I-PACAP1-38

Mice were anesthetized with urethane and administered PACAP1-38 as an eye drop (1 × 10^6^ cpm/eye in benzalkonium-chloride). Serum, whole brain, and eyes were collected 10 or 60 min after the eye drops were administered. The whole brain and eyes were washed in ice-cold PBS. Blood was allowed to clot, centrifuged at 5400× *g* for 10 min, and 50 µL of the serum obtained was added to 200 µL of 1% BSA in Lactated Ringers’ (LR) solution. This mixture was added to 250 µL of 30% trichloroacetic acid, vortexed, and centrifuged for 10 min at 5400× *g*. Brains and eyes were homogenized separately in 0.5 mL of 1% BSA-LR with a bead beater for 30 s at 4800 rpm × 2 on ice. Samples were transferred to a microfuge tube and centrifuged at 10,000× *g* for 20 min and a portion of the resulting supernatant added to an equal volume of 30% trichloroacetic acid, vortexed, and centrifuged at 5400× *g* for 10 min. Resulting supernatant (S) and precipitate (P) from the serum, whole brain, and eyes were counted separately and the percent of radioactivity which could be precipitated with acid (% Precip) was calculated using: % Precip = 100(P)/(S + P).

To correct for any degradation that might have occurred during the processing for acid precipitation, we added 125I-PACAP1-38 to non-radioactive arterial whole blood, whole brain, and eyes, and processed as above. Biological samples were corrected for degradation during processing by dividing their values by the processing control values.

### 4.5. Measurement of Glial Fibrillary Acidic Protein (GFAP) Activity in the Müller Glial Cells

For immunohistochemical analysis, 2 weeks after the induction of ischemia, animals (*n* = 4 sham, *n* = 4 sham + PACAP1-38 and *n* = 4 BCCAO; *n* = 4 BCCAO + PACAP1-38 retinas) were sacrificed with 120 mg/kg pentobarbital (Nembutal, Sanofi-Phylaxia, Budapest, Hungary). The eyes were dissected thereafter in ice-cold PBS and fixed in 4% paraformaldehyde dissolved in 0.1 M PB (pH 7.4) for 4 h at room temperature, similarly to earlier descriptions [35]. Retinas were washed in 0.1 M PB (6 × 10 min) and cryoprotected in 10% and 20% sucrose for 1 h, followed by 30% sucrose in PBS (Sigma, Budapest, Hungary) overnight at 4 °C. Tissues were embedded in tissue freezing medium for cryostat sectioning (Cryomatrix, Shandon, Waltham, MA, USA). Ten–twelve µm setions were made with a cryostat (Leica, Nussloch, Germany). Central retinal areas within 2 mm from the optic nerve head were used for immunohistochemical analysis. Retina sections were mounted on chrome–alum–gelatin coated slides and stored at −20 °C until use. Sections were rinsed in PBS, permeabilized by incubation for 6 × 5 min in 0.1% Triton X-100 (Sigma, Budapest, Hungary) in PBS and incubated with 3% normal donkey serum and 0.1% Na-azide in PBS for 1 h to minimize nonspecific labeling. Incubation with the primary polyclonal antibody, anti-GFAP, lasted overnight at 4 °C (rabbit anti-GFAP 1:1000, Sigma), in 1% donkey serum (Sigma). After being washed in PBS six times, incubation in the secondary fluorescent anti-rabbit antibody Alexa Fluor 488 (donkey anti-rabbit, 1:200, Life Technologies, Budapest, Hungary) followed for 2 h at room temperature in the dark. After washing in PBS, propidium iodide (PI, 1:500, Sigma) was used to detect the nuclear components. After three washes in PBS, sections were coverslipped using Fluoroshield (Sigma). Primary antibodies were omitted for control experiments, resulting in no specific staining. Digital photographs were taken with a Nikon Eclipse Ci fluorescence microscope. Photographs were further processed with the Adobe Photoshop CS6 program (version 13.0 X64, San Jose, CA, USA). Images were adjusted for contrast only; they were aligned, arranged and labeled by using the functions of the above program. The examiner evaluating the images was not aware of the experimental groups.

### 4.6. Statistical Analysis

Statistical comparisons were made using the two-way ANOVA followed by Fischer’s post hoc analysis.

## 5. Conclusions

Based on the present results, we conclude that PACAP1-38, delivered as an eye drop, effectively ameliorates the effects of rat chronic retinal ischemia.

## Figures and Tables

**Table 1:** Acid precipitation of radioactivity extracted from eyes, brain, and serum after ocular administration of PACAP1-38 labeled with radioactive iodine. Percentage of acid precipitation for eyes, whole brain, and serum taken at 5 and 60 min post ocular administration of ^125^I-PACAP1-38. Values are corrected for degradation that occurred during processing. Results are means ± SEM from an *n* = 3 at each time point.

Region of Interest	10 min	60 min
Eye	52.73 ± 2.35	48.24 ± 3.39
Whole Brain	104.71 ± 17.72	75.09 ± 41.13
Serum	25.57 ± 25.57	0 ± 0
